# A Rare Case of Pneumococcal Appendicitis in a Child

**DOI:** 10.1155/2022/9262149

**Published:** 2022-02-07

**Authors:** Amar Chikhaoui, Néhémie Nzoyikorera, Mehdi El Mouadden, Mounia Al Zemmouri, Khalid Zerouali

**Affiliations:** ^1^Faculty of Medicine and Pharmacy of Casablanca, Hassan II University of Casablanca, Casablanca, Morocco; ^2^Bacteriology, Virology and Hospital Hygiene Laboratory, Ibn Rochd University Hospital, Casablanca, Morocco; ^3^Pediatric Visceral Surgery, Ibn Rochd University Children's Hospital, Casablanca, Morocco

## Abstract

Appendicitis is the most common cause for abdominal surgery in children. It is usually caused by *Escherichia coli* and *Streptococcus* species and is generally polymicrobial. However, *Streptococcus pneumoniae* is a rare cause of appendicitis. We report a rare case of pneumococcal appendicitis in a 7-year-old child with no underlying conditions, in association with *E. coli* and group F *β*-hemolytic *Streptococcus*. The isolated pneumococcal strain was sensible to all tested antibiotics. The patient had a full recovery after surgery and antibiotics. This case emphasizes that *S. pneumoniae* can cause a variety of unusual infections like appendicitis, in patients with or without underlying conditions. Thus, even though being a rare entity, physicians should always be aware of *S. pneumoniae* as a possible causative agent.

## 1. Introduction

*Streptococcus pneumoniae* (*S. pneumoniae*) can cause a wide spectrum of infections like meningitis, pneumonia, bacteremia, and otitis. Furthermore, it has been reported that the pneumococcus can demonstrate very atypical presentations, like cardiovascular, genitourinary tract, and gastrointestinal infections [1]. We report a rare case of *S. pneumoniae* causing appendicitis in a child.

## 2. Case Presentation

A 7-year-old male child presented at Ibn Rochd University Children's Hospital with a history of abdominal pain localized in the right lumbar region, fever, and food emesis for two days. No intestinal transit disorders were reported. Abdominal examination showed right lower quadrant abdominal pain (positive McBurney sign) and no signs of peritoneal irritation. Rovsing, Blumberg, and Psoas signs were negative. The patient had a fever of 38.6°C. There were no other pathological findings on physical examination.

Laboratory analysis highlighted a white blood cell count of 10.2 × 10^3^/mm^3^ (83% of neutrophils) and a C-reactive protein level of 272.8 mg/L. No other biological or radiological exams were done as the patient was directly taken to the operating room. The final diagnosis was acute appendicitis.

The abdomen was opened by McBurney incision. An inflamed, enlarged, and perforated appendix with periappendicular pus was found perioperatively. The rest of the peritoneal cavity was clean. A pus sample was collected, and the appendicectomy was done. The pus was sent to the laboratory for bacteriological analysis.

Gram-stained direct smear showed Gram-positive cocci. The culture was polymicrobial with *S. pneumoniae*, *E.coli*, and group F *β*-hemolytic streptococci. Antimicrobial sensitivity testing was done for *S. pneumoniae* following the EUCAST guidelines. The pneumococcal strain was sensitive to penicillin G, ceftriaxon, erythromycin, tetracycline, co-trimoxazol, and levofloxacin. Serogrouping/serotyping was done by the checkerboard method with Pneumotest-Latex (Statens Serum Institute antisera, Copenhagen, Denmark) and multiplex PCR according to the protocol proposed by the CDC [2], but the serotype has not been identified. Thus, the strain was categorized as a nonvaccine serotype (NVS).

After the procedure, the patient had a full recovery on a treatment consisting of an association of gentamycin 100 mg IV daily for three days, ampicillin-sulbactam 3 g IV daily, and metronidazole 1.2 g IV daily for five days. Oral antibiotic treatment was continued 5 days after discharge.

## 3. Discussion

Intra-abdominal infections and particularly appendicitis are rarely caused by *S. pneumoniae.* The estimated incidence rate of pneumococcal appendicitis is 0.25/100000 [3]; Heltberg et al. reported only six cases of pneumococcal appendicitis from 1967 to 1981 in 2 hospital settings, representing approximately 0.3% of appendicitis patients [4].

The etiology of appendicitis is usually polymicrobial and associated with common intestinal pathogens, which corresponds to our case. However, in most reported cases of pneumococcal appendicitis, *S. pneumoniae* was detected in pure cultures and that make our case a rare occurrence [5–8].

Some hypotheses have been considered to explain the involvement of *S. pneumoniae* in appendicitis. The colonization of the bowel may be the source of the pathologic process, but *S. pneumoniae* is very susceptible to the bactericidal effect of gastric acid. Furthermore, no studies have established the sporadic presence of the pneumococcus in the intestinal flora. The appendix could also be reached by hematogenous spread from the colonized respiratory tract, particularly in children, whose nasopharynxes are densely colonized. It should also be noted that a number of proved appendicitis patients had a history of respiratory symptoms of pneumonia [9].

Few articles report microbiological characteristics in case reports. As in ours, the majority reported *S. pneumoniae* strains that were sensitive to most tested antibiotics [5, 6, 8]. In Casablanca, vaccination has considerably reduced the prevalence of multiresistant serotypes, which were often associated with some of the prevaccination serotypes covered by PCV, such as 9V, 6B, 14, 19A, 19F, and 23F [10]. However, only a handful reported the serotypes of the isolated strains: serogroup 19 was the most prevalent followed by serotype 3 [5]. Concerning our case, a NVS was found.

The most frequent risk factors associated with invasive pneumococcal disease include patients with cardiopathy, chronic lung disease, diabetes, immunosuppression (nephrotic syndrome, malignancies, etc.), and splenic dysfunction (splenectomy, asplenia, etc.) [11]. However, in our case study, none of these factors were present and the vaccination status was unknown. Male gender could be the only risk factor in our case, which is documented in the literature [12]. This concords with findings from other case reports where most patients had no predisposing factors for pneumococcal infections [4, 5].

According to the World Journal of Emergency Surgery guidelines, triple agent antibiotic therapy should be administered after appendicectomy in cases of perforated appendicitis in children. Several options are possible, one of which is the association of ampicillin-metronidazole and gentamycin, which was used in our case. Alternatives include ceftriaxone-metronidazole or ticarcillin-clavulanate plus gentamicin, in accordance with the epidemiology of bacteria [13]. The duration of the treatment ought to be shorter than 7 days, and transition to oral therapy should be done as soon as possible, since oral antibiotics show equivalent outcomes compared with intravenous therapy, but with shorter length of hospitalizations [13].

In our case report, the patient had a full recovery with no complications. This concords with reported cases like the case of Ghadage [6]. Heltberg et al. reported 2 patients who presented mild complications while Dimond and Proctor reported a particular case of a 9-year-old patient who presented pneumococcal meningitis postoperatively from a peritonitis secondary to an appendicitis [5, 14].

## 4. Conclusion

We reported a rare case of pneumococcal appendicitis in a child without any predisposing factors. A positive outcome was observed after surgery and antibiotic therapy. Although uncommon, *S. pneumoniae* can also cause appendicitis and thus should be considered as a probable cause by physicians in abdominal infections.

## Data Availability

The data used in this article can be found in the archives of the Pediatric Visceral Surgery Department of Ibn Rochd University Hospital.
